# Supplementary material for: Proposed Mechanism for Emodin as Agent for Methicillin Resistant Staphylococcus Aureus: In Vitro Testing and In Silico Study
Source: Curr Issues Mol Biol. 2022 Sep 27;44(10):4490–9. doi: 10.3390/cimb44100307 (PMC9600265; doi:10.3390/cimb44100307)
Supplement: Supplementary file 1 [file cimb-44-00307-s001.zip › cimb-1926674-supplementary.pdf]

Proposed Mechanism for Emodin as Agent for Methicillin Resistant Staphylococcus Aureus: In Vitro Testing and In Silico Study

Mohammed M. Ghoneim [1,2,†], Rasha Hamed Al-Serwi [3,†], Mohamed El-Sherbiny [4], El-sayed M. El-ghaly [1], Amal E. Hamad [5], Mohamed A. Abdelgawad [6], Ehab A. Ragab [1], Sarah I. Bukhari [7], Khulud Bukhari [8], Khaled Elokely [9,*] and Manal A. Nael [9,10,*]

[1] Pharmacognosy and Medicinal Plants Department, Faculty of Pharmacy, Al-Azhar University, Cairo 11884, Egypt
[2] Department of Pharmacy Practice, College of Pharmacy, AlMaarefa University, Riyadh 11597, Saudi Arabia
[3] Department of Basic Dental Sciences, College of Dentistry, Princess Nourah bint Abdulrahman University, Riyadh 11671, Saudi Arabia
[4] Department of Basic Medical Sciences, College of Medicine, AlMaarefa University, Riyadh 11597, Saudi Arabia
[5] Department of Pharmaceutical Analytical Chemistry, Faculty of Pharmacy, Menoufia University, Shebin El-Koum 32511, Egypt
[6] Department of Pharmaceutical Chemistry, College of Pharmacy, Jouf University, Sakaka 72341, Saudi Arabia
[7] Department of Pharmaceutics, College of Pharmacy, King Saud University, Riyadh 11451, Saudi Arabia
[8] Department of Microbiology and Parasitology, College of Veterinary Medicine, King Faisal University, Hofuf, Al-Ahsa 36362, Saudi Arabia
[9] Department of Chemistry, Institute for Computational Molecular Science, Temple University, Philadelphia, PA 19122, USA
[10] Department of Pharmaceutical Chemistry, Faculty of Pharmacy, Tanta University, Tanta 31527, Egypt
* Correspondence: kelokely@temple.edu (K.E.); manal.nael@temple.edu (M.A.N.)
† These authors contributed equally to this work.

Table of Content

	Page
**Figure S1. ^{1}H NMR spectrum of compound 1.**	**3**
**Figure S2.^{13}C NMR spectrum of compound 1.**	**4**
**Figure S3. HRESIMS (-) for compound 1.**	**5**
**Figure S4. ^{1}H NMR spectrum of compound 2.**	**6**
**Figure S5. ^{13}C NMR spectrum of compound 2**	**7**
**Figure S6. HRESIMS (-) for compound 2**	**8**
**Figure S7. Emodin contacts with amino acids in site 2**	**9**
**Figure S8. Average contacts of emodin in site 2**	**10**
**Figure S9. Emodin contacts with amino acids in site 3**	**11**
**Figure S10. Average contacts of emodin in site 3**	**12**

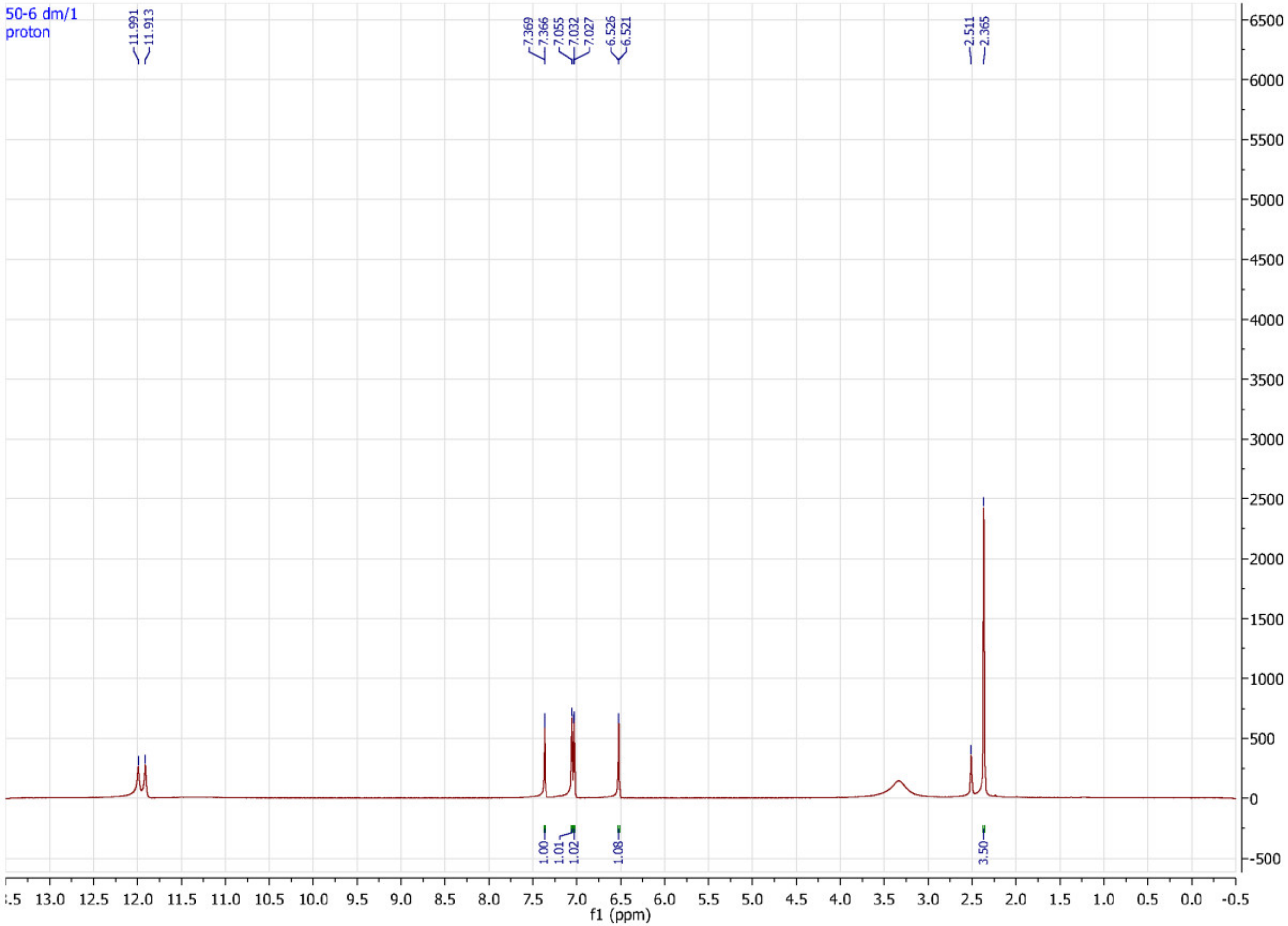

Figure S1. ^{1}H NMR spectrum (400 MHz) for compound **1** in DMSO-d_6.

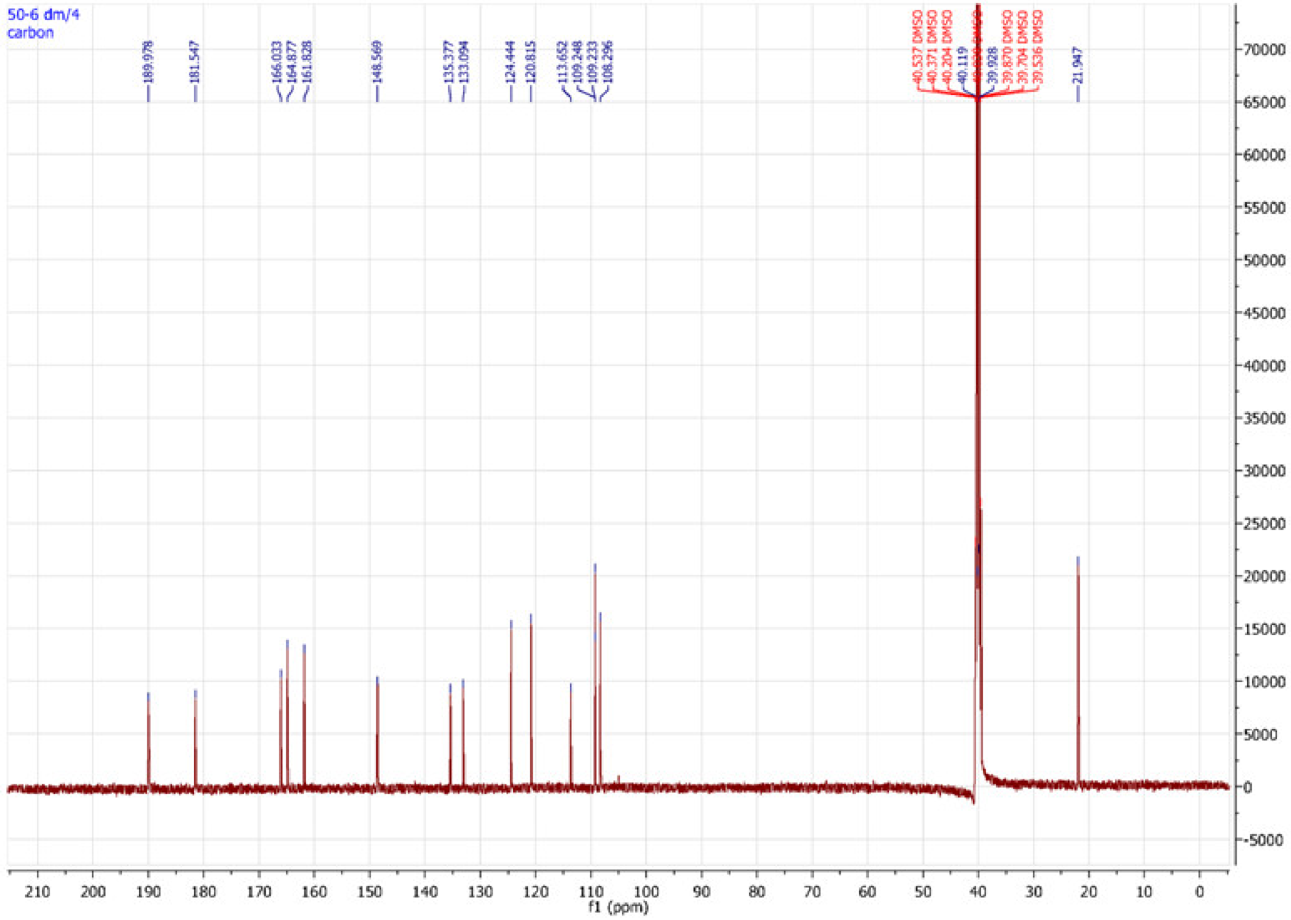

Figure S2. ^{13}C NMR spectrum (400 MHz) for compound **1** in DMSO-d_6

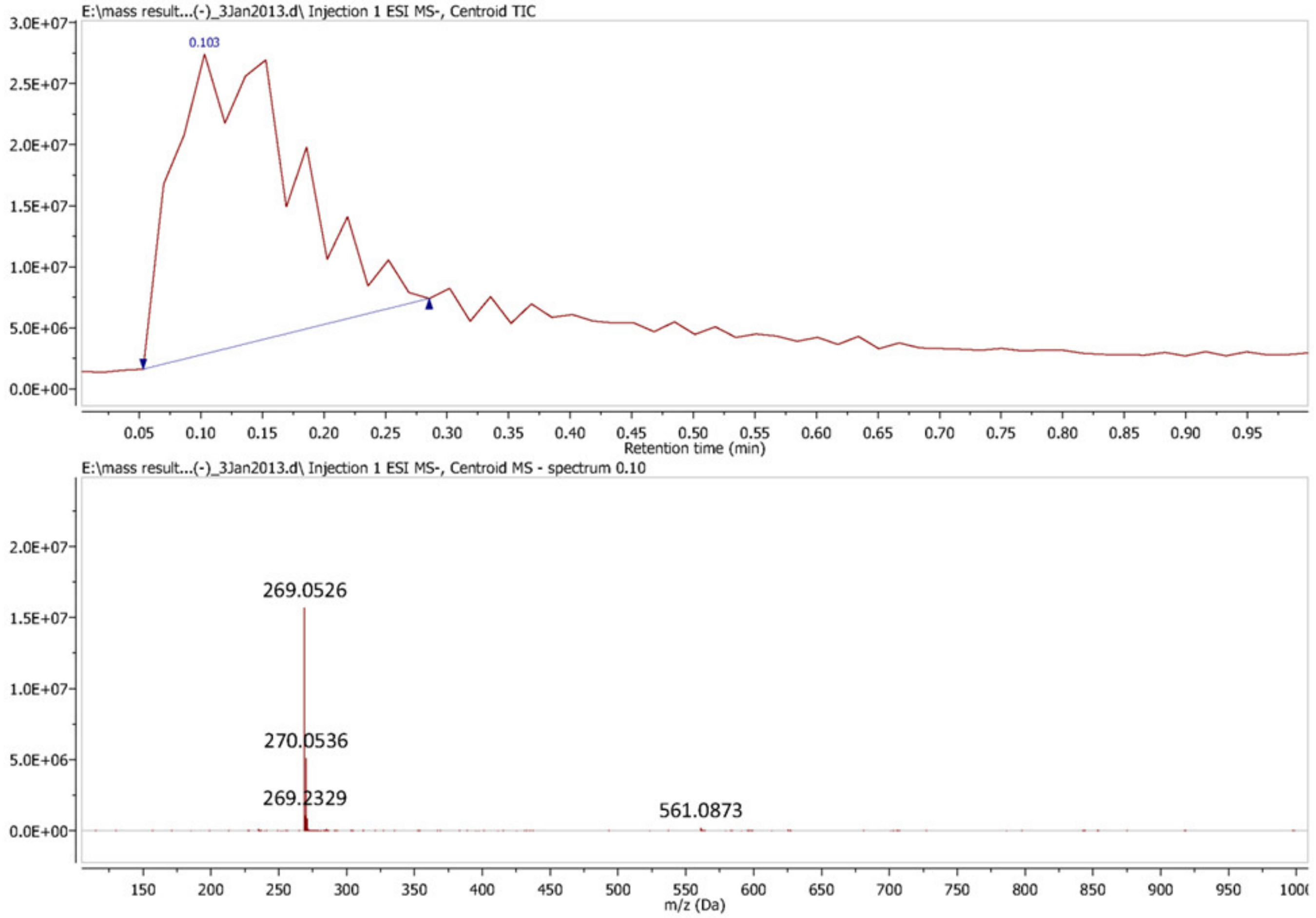

Figure S3. Negative ESI/MS spectrum for compound **1**

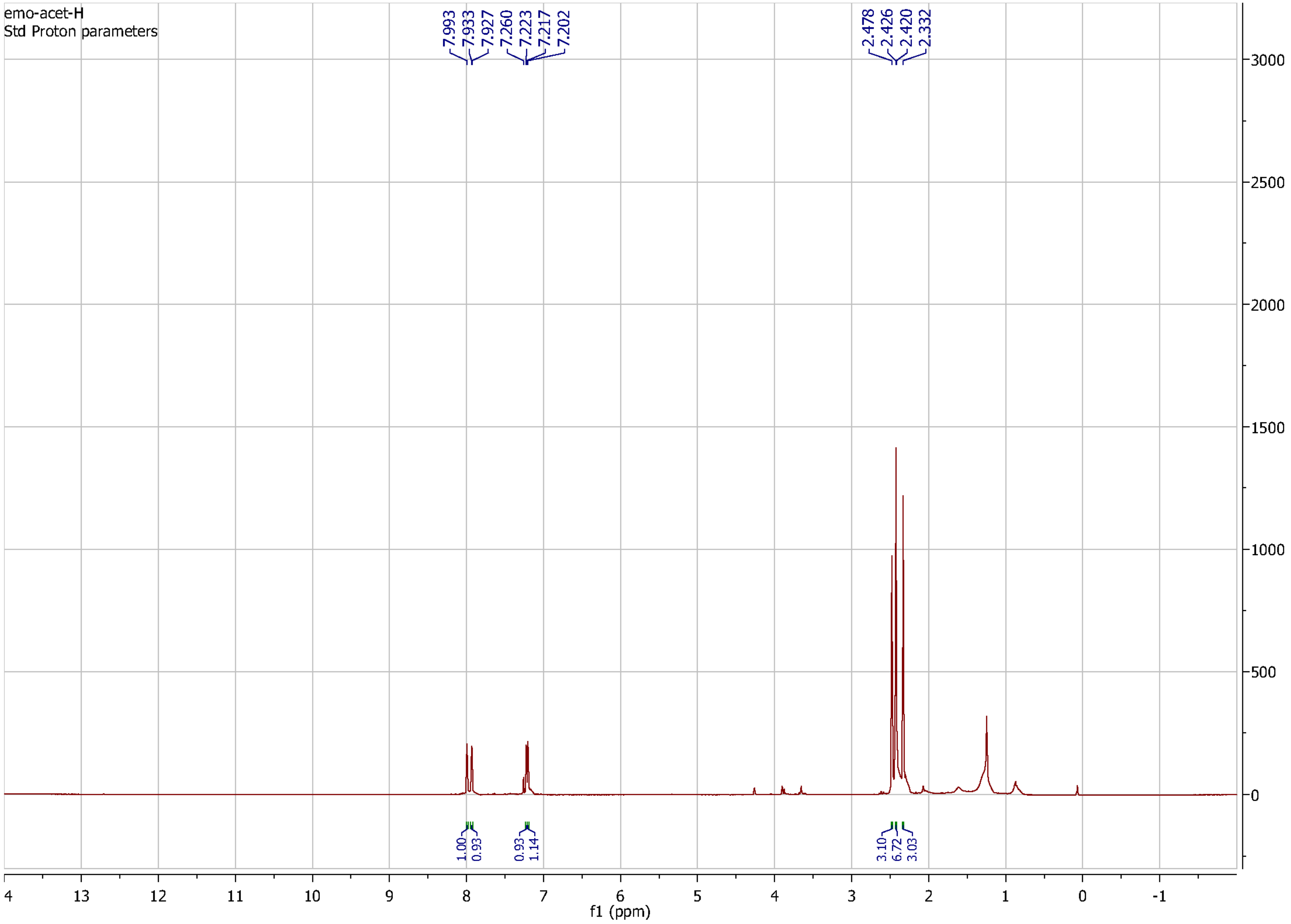

Figure S4. ^{1}H NMR spectrum (400 MHz) for compound **2** in $CDCL_3$-*d*.

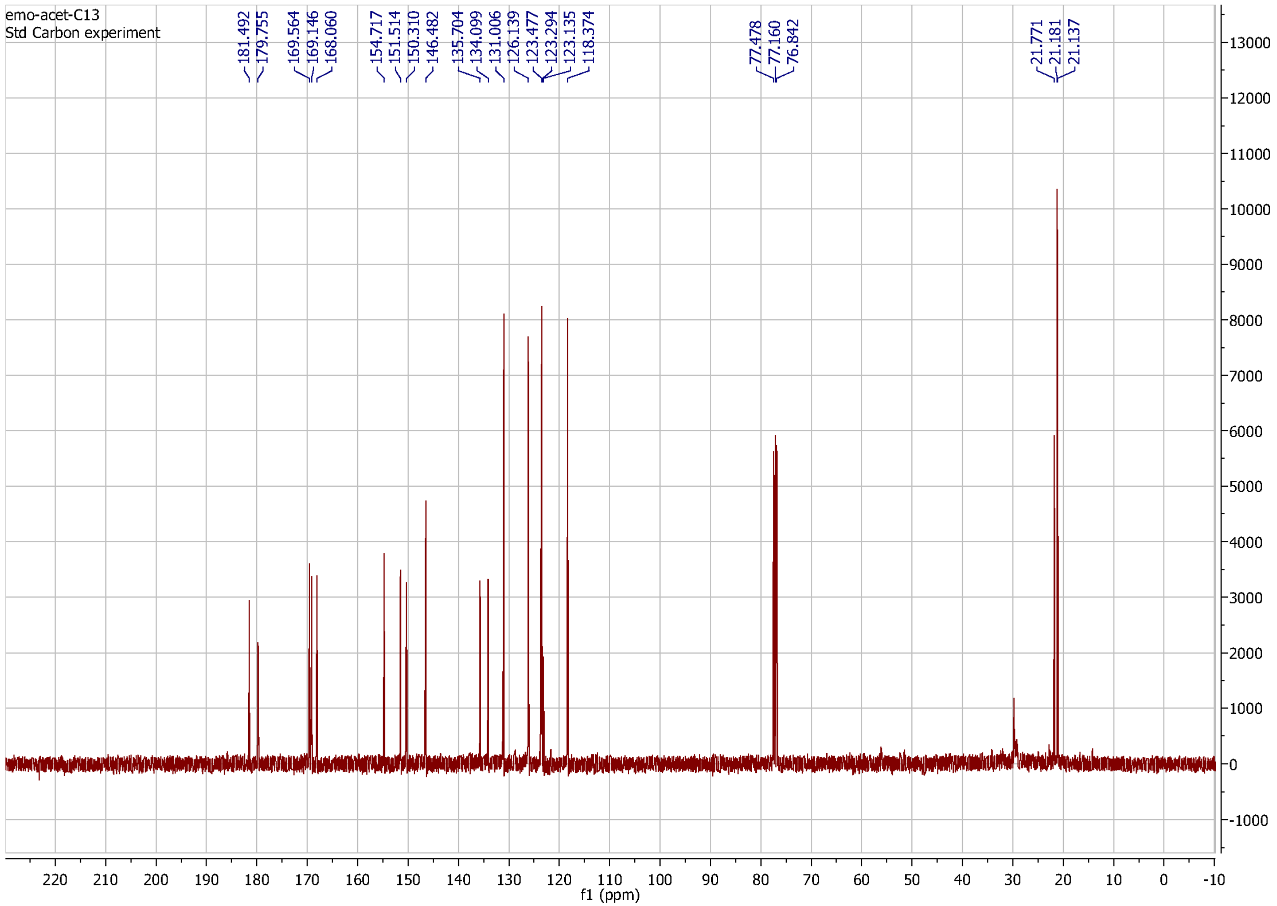

Figure S5. ^{13}C NMR spectrum (400 MHz) for compound **2** in $CDCL_3$-*d*

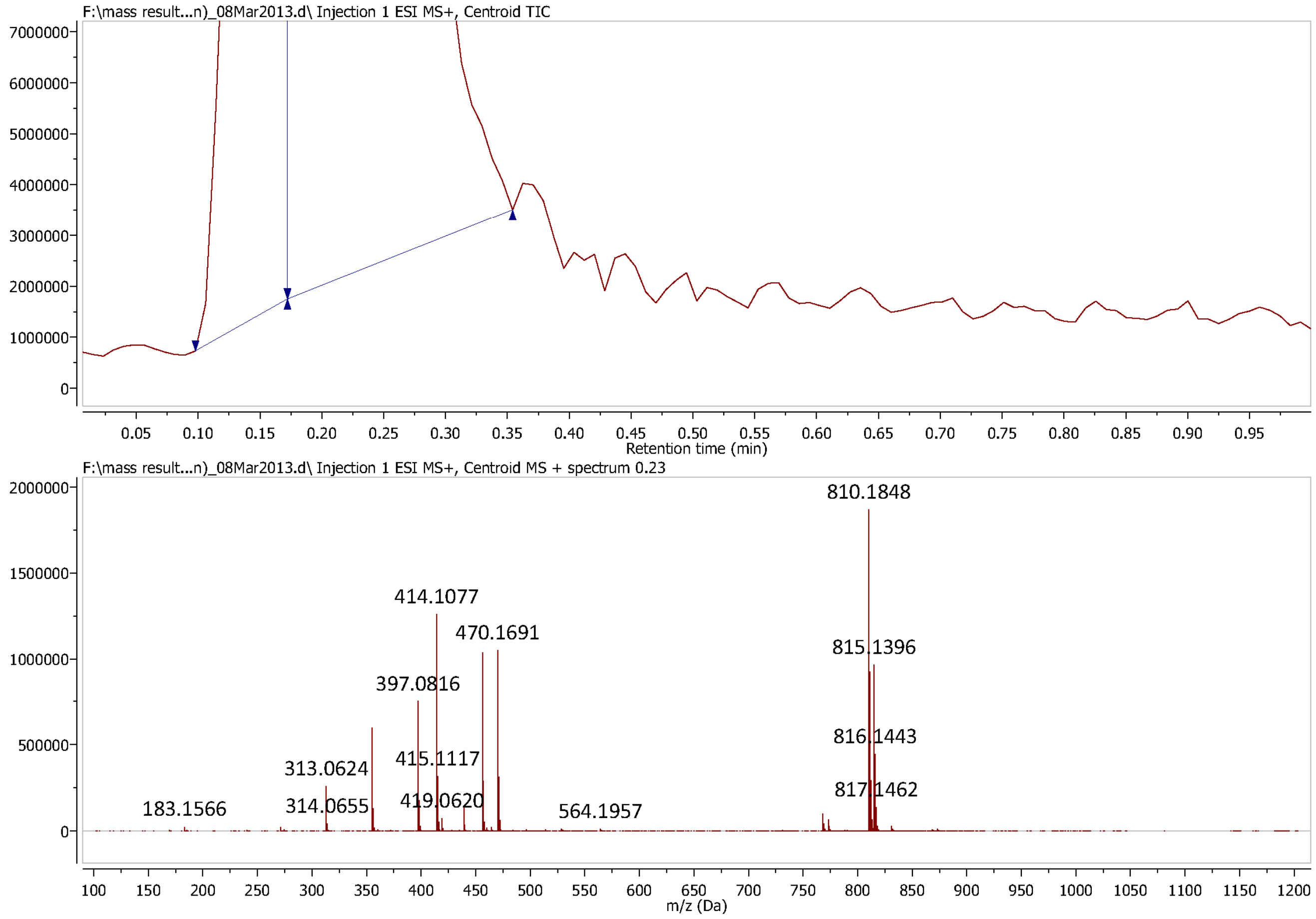

Figure S6. Positive ESI/MS spectrum for compound **2**

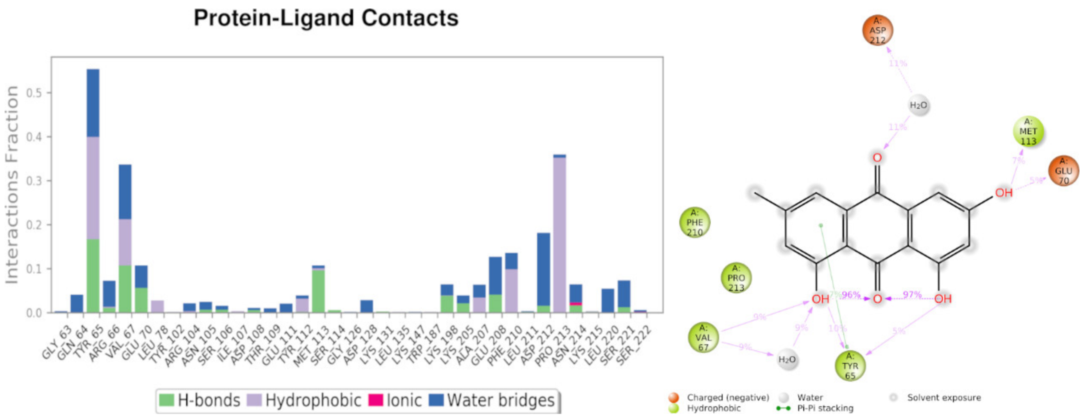

Figure S7. Emodin contacts with amino acids in site 2.

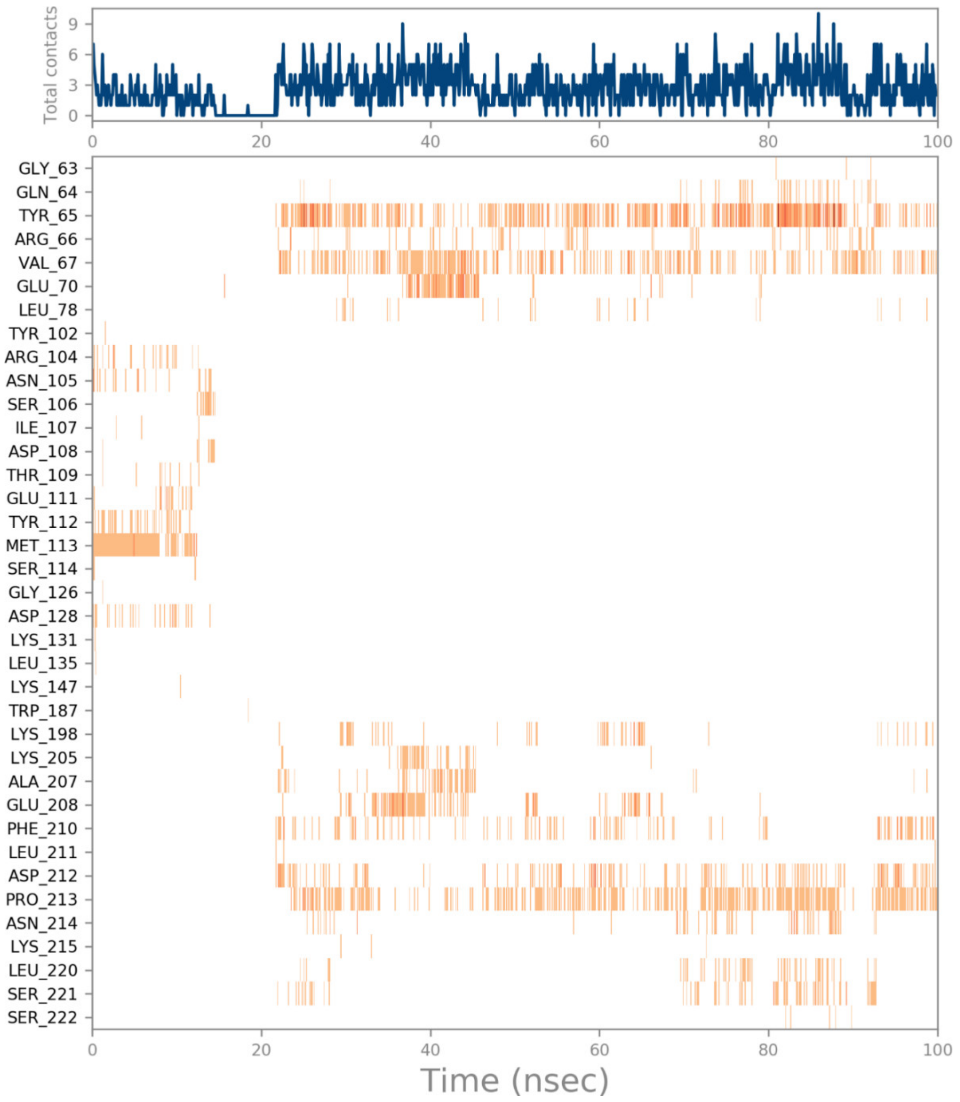

Figure S8. Average contacts of emodin in site 2.

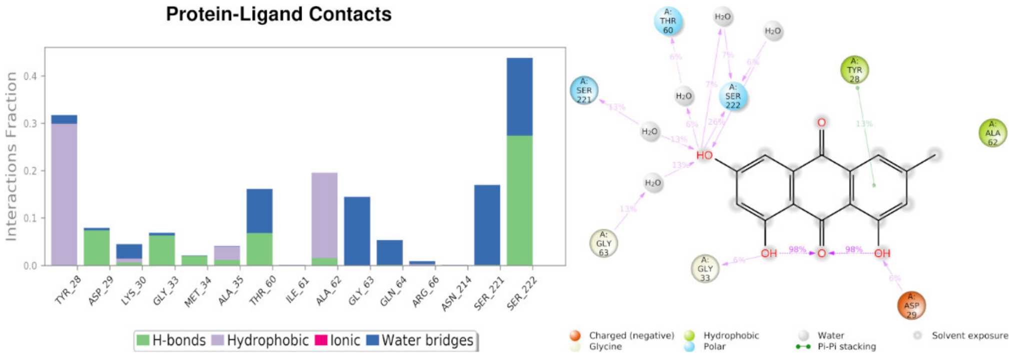

Figure S9. Emodin contacts with amino acids in site 3.

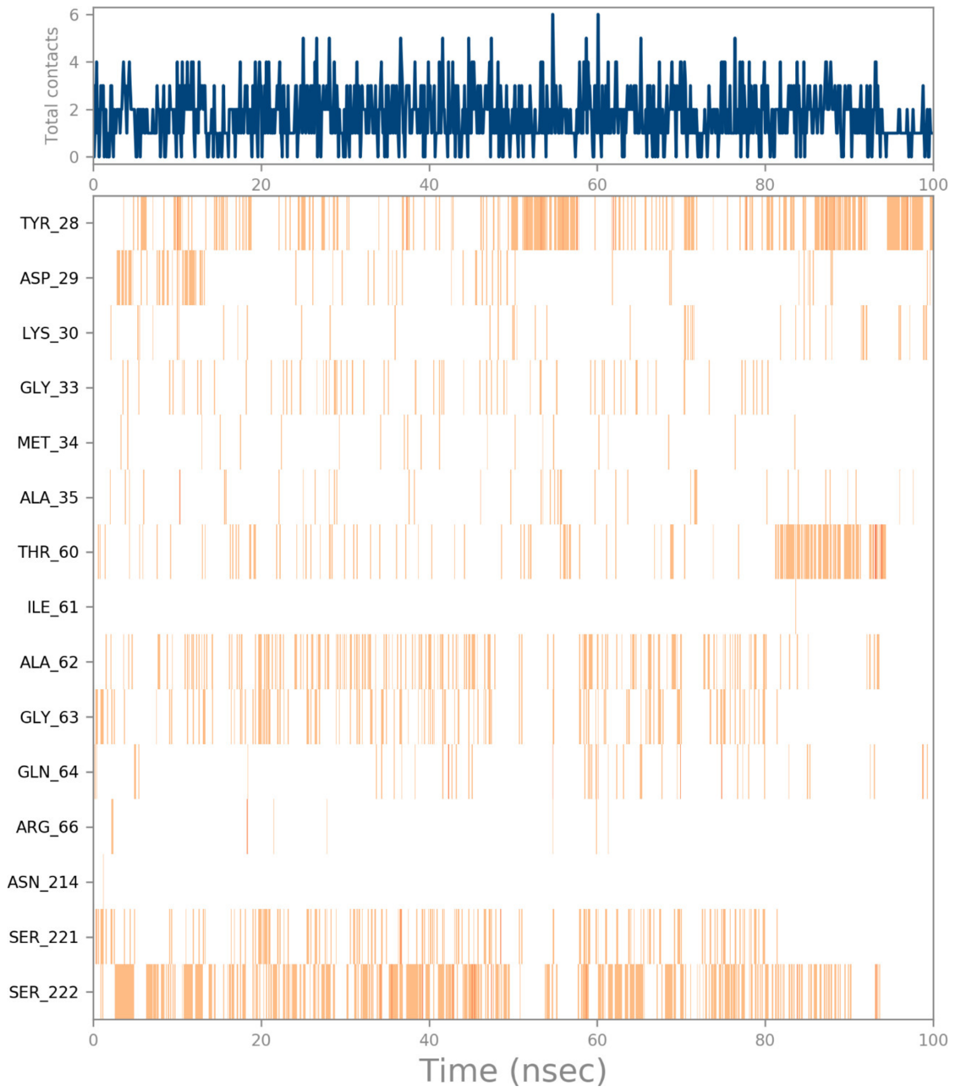

Figure S10. Average contacts of emodin in site 3.
